# Long-term prognosis and clinical course of choking-induced cardiac arrest in patients without the return of spontaneous circulation at hospital arrival: a population-based community study from the Shizuoka Kokuho Database

**DOI:** 10.1186/s12873-022-00676-8

**Published:** 2022-07-06

**Authors:** Takahiro Miyoshi, Hideki Endo, Hiroyuki Yamamoto, Koki Shimada, Hiraku Kumamaru, Nao Ichihara, Yoshiki Miyachi, Hiroaki Miyata

**Affiliations:** 1 Shizuoka Graduate University of Public Health, 4-27-2 Kita Ando Aoi-ku, Shizuoka City, 420-0881 Japan; 2 Department of Health Policy and Management, School of Medicine, Keio University, Tokyo, Japan; 3 Department of Healthcare Quality Assessment, The University of Tokyo Graduate School of Medicine, Tokyo, Japan

**Keywords:** Choking, Cardiac arrest, Resuscitation, Long-term prognosis, Clinical course

## Abstract

**Background:**

The risk of choking increases with aging, and the number of cases of choking-induced cardiac arrest is increasing. However, few studies have examined the prognosis of choking-induced cardiac arrest. The aim of this study was to reveal the rates of survival and dependence on devices in the long term after choking-induced cardiac arrest.

**Methods:**

We analyzed data from the Shizuoka Kokuho Database, which consists of claims data of approximately 2.2 million people, from April 2012 to September 2018. We selected patients with choking-induced cardiac arrest who received cardiopulmonary resuscitation in the hospital. Patients were excluded if they were less than 20 years old, had an upper airway tumor, received ventilation assistance, or received enteral nutrition in the month prior to cardiac arrest. The primary outcome was death, and the secondary outcomes were the rates of survival at 3-months and independence on devices. Descriptive statistics are presented and compared among age groups (20–64 years, 65–74 years, 75–84 years, 85 years and older), and survival time analysis (Kaplan-Meier method) was performed.

**Results:**

In total, 268 patients were analyzed, including 26 patients in the 20–64 age group, 33 patients in the 65–74 age group, 70 patients in the 75–84 age group, and 139 patients in the ≥85 age group. The overall 3-month survival rate was 5.6% (15/268). The 3-month survival rates were 3.8% (1/26) in the 20–64 age group, 15.2% (5/33) in the 65–74 age group, 8.6% (6/70) in the 75–84 age group, and 2.2% (3/139) in the ≥85 age group. The overall 12-month survival rate was 2.6% (7/268). Of the 7 patients who survived for 12 months, 3 received ventilation management and 5 received tube or intravenous feedings at 3 months. These survivors were still receiving ventilation assistance and tube feedings in the hospital and had not been discharged at 12 months.

**Conclusions:**

The prognosis of choking-induced cardiac arrest was extremely poor when patients were not resuscitated before hospital arrival. Those who survived were mostly dependent on assistive devices. Additionally, none of the survivors dependent on assistive devices had discontinued the use of the devices at the long-term follow-up.

**Supplementary Information:**

The online version contains supplementary material available at 10.1186/s12873-022-00676-8.

## Introduction

Choking is an emergency condition that can lead to death if the responsible object is not removed quickly. The number of deaths from asphyxia due to choking increases with age in the elderly population [1–6]. In Japan, where the aging population is increasing at the fastest rate in the world, there are approximately 9000 deaths due to choking annually, making it the second most common cause of accidental death [3]. Considering the aging of populations, especially in developed countries, it is expected that choking-induced cardiac arrest will become a major problem in the future [1–6].

Little research on the prognosis after choking-induced cardiac arrest has been performed. In one of the few studies on choking-induced cardiac arrest, Igarashi et al. reported a survival discharge rate of 28%, with only 5% of patients having a good neurological prognosis (cerebral performance categories (CPCs): 1–2) at the time of discharge [7]. However, this was a single-center study, which limited its generalizability. Furthermore, the long-term prognosis for choking-induced cardiac arrest has not been evaluated.

In a study of 210,642 cardiogenic out-of-hospital cardiac arrest (OHCA) patients, Suematsu et al. reported that the 30-day survival rate was 9.6% and that the proportion of patients with a good neurologic prognosis at 30 days was 5.6% [8]. Regarding long-term prognosis, in a Norwegian study in patients admitted to a hospital for cardiogenic OHCA, Kvakkestad et al. reported that the 8-year survival rate was 49% [9]. In an Australian study of 2983 surviving discharged cardiogenic OHCA patients, Andrew et al. reported that the survival rates at 1 and 5 years were over 90% and over 80%, respectively [10]. According to the above study, if a patient with cardiogenic OHCA survives to admission, their prognosis is expected to be good in the long term. However, the physiological mechanism of choking-induced cardiac arrest differs from that of cardiogenic cardiac arrest, and its prognosis may be different. No studies have been conducted on this topic.

It is expected that some discharged patients will be device-dependent, and this will impose financial and emotional burdens on their families or partners [11, 12]. Therefore, research on the status of discontinuation of assistive devices after discharge can provide a basis for families and physicians for the establishment of a treatment plan. In addition, another study in patients who received cardiopulmonary resuscitation for any cause reported that in the long-term, “cognitive function” and “activities of daily living (ADL)” improved for up to 12 months after resuscitation [13, 14]. Therefore, long-term follow-up of patients with choking-induced cardiac arrest will help clarify improvements in living conditions improve and guide the decision to aggressively intervene during treatment for choking-induced cardiac arrest.

In this study, we revealed the rates of survival and dependence on devices at 3 and 12 months. Based on the results, we evaluated the long-term prognosis and clinical course of choking-induced cardiac arrest without ROSC at hospital arrival.

## Methods

### Database

We analyzed data from the Shizuoka Kokuho Database in this study [15–18]. This database is owned by the Federation of National Health Insurance Organizations and consists of administrative claims data from Shizuoka Prefecture, Japan. Shizuoka Prefecture had a population of approximately 3.6 million in 2020.

There are three main types of health insurance plans in Japan: Employee’s Health Insurance (EHI), National Health Insurance (NHI), and Late Elder’s Health Insurance (LEHI); EHI and NHI are for those under the age of 75. NHI is for people who are mainly self-employed citizens, part-time workers, or unemployed. Employees of large or small companies are not enrolled in the NHI plan. All individuals over the age of 75 are enrolled in the LEHI plan. The Shizuoka Kokuho Database consists of NHI and LEHI data and covers approximately 2.2 million people in Shizuoka Prefecture [18].

In this study, we analyzed data from April 2012 to September 2018. The data included registrant information (including age, sex, observation period, reason for withdrawal, and death dates), insurance claim data (including prescribed medicines, procedures, and 10th Revision of the International Classification of Diseases (ICD-10) codes), and the long-term care insurance data (including support and care level as well as information about care services provided for insured individuals). These claims data were updated on a monthly basis. The ICD-10 diagnostic codes were updated for the duration of treatment for a specific disease. All prescribed medicines are coded in Japanese original codes, and each code is linked to an Anatomical Therapeutic Chemical Classification System (ATC) code. These data were tied to individuals by anonymized individual identifiers for research purposes.

This study was approved by the institutional review board of Shizuoka General Hospital (Shizuoka, Japan; SGHIRB#2020021), and informed consent was waived because the data were anonymized.

### Study population

We included those with cardiac arrests without return of spontaneous circulation (ROSC) on arrival at the hospital and assumed that all cardiac arrest patients who arrived at the hospital without ROSC received closed chest compressions after arrival at the hospital. To identify people with cardiopulmonary arrests without ROSC on arrival at the hospital, we first included those who had a record of closed chest compressions in the admission month. We excluded those who were less than 20 years old with cardiac arrests due to causes other than choking. The cause of cardiac arrest was determined to be choking by the presence of the ICD-10 codes for choking (codes: T172, T173, T174, T175, T178, T179, T71). We excluded those receiving ventilation assistance or who received enteral nutrition in the month prior to cardiac arrest because they were not living independently and may have had serious dysphagia before the event. We excluded those with an upper airway tumor because their upper airway may have been restricted by the tumor and the tumor may have affected their long-term prognosis. We excluded those who lacked claims data during the 6 months before the event because we could not evaluate information about comorbidities and medications before the event.

### Outcomes

We defined the primary endpoint as death, and survival rates were analyzed at 3 and 12 months. In this database, deaths were recorded separately from insurance withdrawal. We defined the secondary endpoint as independence on tube feeding or total parenteral nutrition (TPN) at 3 and 12 months.

### Definitions and data collection

Other variables included age, sex, comorbidities (dementia, stroke, Sjogren’s syndrome), medications (antipsychotics, dopamine agonists, sedative agents, anticholinergic agents), and living conditions before the event (ADL, nursing home residency, history of aspiration pneumonia, dysphagia rehabilitation). In addition, we considered targeted temperature management, tracheostomy, gastrostomy, and dysphagia rehabilitation as procedures after choking-induced cardiac arrest. From the ICD-10 diagnostic codes registered within 12 months prior to choking, we identified comorbidities (stroke, Sjogren’s syndrome, aspiration pneumonia). From the ATC codes, we identified medicines (donepezil, antipsychotics, dopamine agonists, sedative agents, anticholinergic agents) prescribed prior to choking. We defined dementia as the use of donepezil. From the long-term care insurance data, we identified the level of care needed and nursing home admission. In Japan, level of care is categorized into seven levels: two levels of support, with relative independence; and five levels of nursing care [19, 20]. We defined independence in ADL as level 2 or lower care and dependence as level 3 or higher care. From the records of procedures after choking in the insurance claims database, we identified dysphagia rehabilitation, targeted temperature management, tracheostomy, and gastrostomy.

### Statistical analysis

We summarized data as the mean and standard deviation (SD) or median and interquartile range (IQR) for continuous variables and frequency and percentage for categorical variables. Comparisons were made using the Mann-Whitney U test for continuous variables and the χ2 test or Fisher’s exact test for categorical variables.

Kaplan-Meier survival analysis was used to assess long-term survival. Survival time was compared among age groups (20–64 years, 65–74 years, 75–84 years, 85 years and older). It was also compared between the ventilator- and nonventilator-dependent groups at 3 months.

Statistical analyses were performed using Stata (version: 16.1; StataCorp, 2020, College Station) and R (version: 4.0.1; R Foundation for Statistical Computing), and a two-sided significance level < 0.05 was considered statistically significant.

## Results

**Fig. 1:**
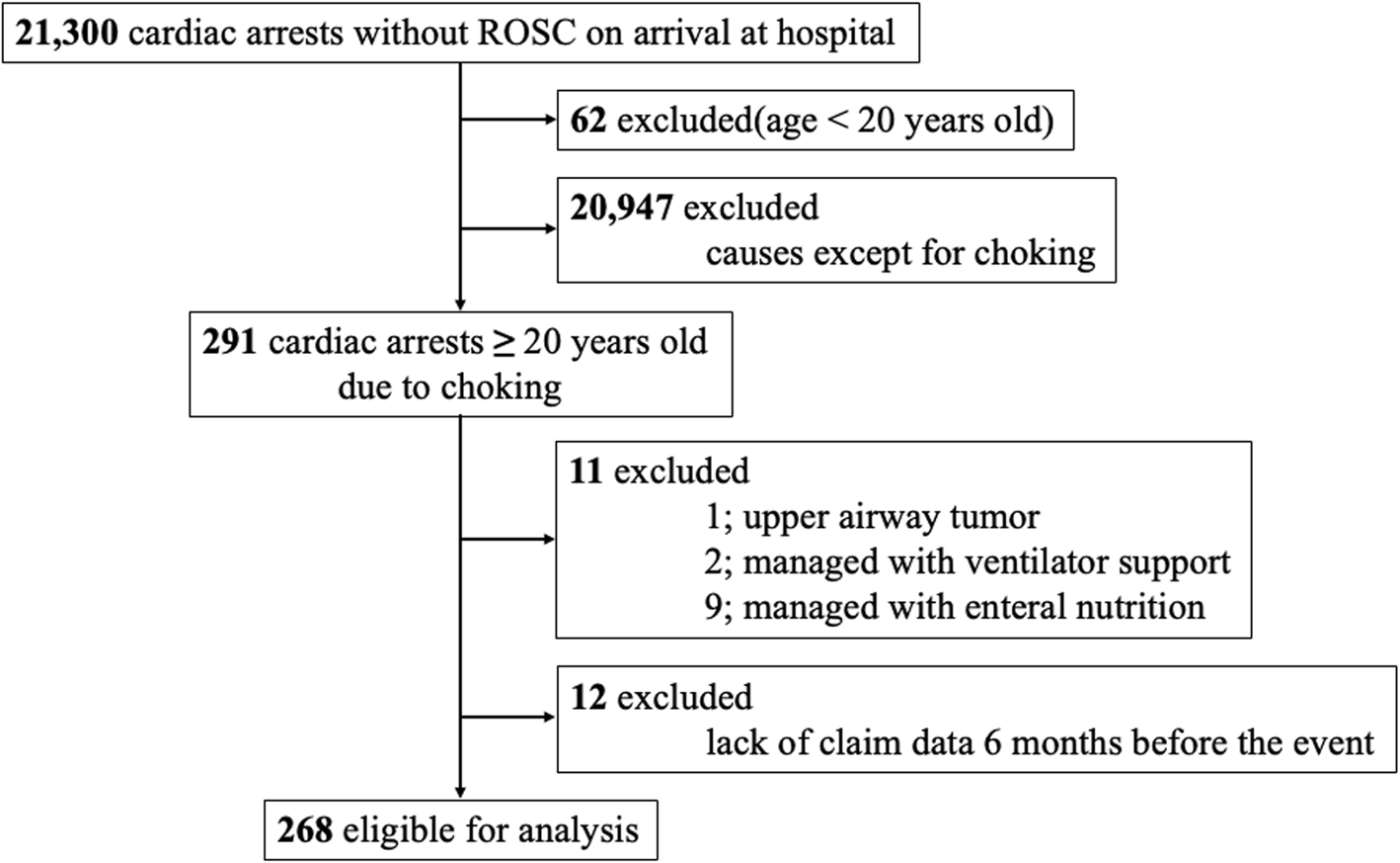
Flow diagram showing of the inclusion-exclusion process

A total of 21,300 cardiac arrests were identified in the Shizuoka Kokuho Database from April 2012 to September 2018. Of these cases, 268 patients were included in the final analysis (Fig. 1).

### Comparisons among age groups

The characteristics of the patients with choking-induced cardiac arrest by age group are shown in Table 1. More than half of the individuals who experienced choking-induced cardiac arrest were over 85 years old. The numbers of patients with antipsychotics and sedative agents use tended to be higher in the younger age group. In the 20–64 age group, 57.7% used antipsychotics, and 50.0% used anxiolytics. The proportion of patients with dopamine agonist use was higher in the 75–84 age group than in the ≥85 age group (≥85 age group: 7.2%, 75–84 age group: 18.6%), and the proportion of patients with stroke was also higher in the 75–84 age group than in the ≥85 age group (≥85 age group: 31.7%, 75–84 age group: 40.0%). Overall, the percentage of patients with Sjogren’s syndrome was 1.9%. The percentage of those with dysphagia rehabilitation within 12 months before choking-induced cardiac arrest tended to increase with age (20–64 age group: 3.8%, 65–74 age group: 0%, 75–84 age group: 5.7%, ≥85 age group: 6.5%). The proportion of patients with a history of aspiration pneumonia within 12 months before choking-induced cardiac arrest tended to increase with age (20–64 age group: 3.8%, 65–74 age group: 3.0%, 75–84 age group: 7.1%, ≥85 age group: 10.1%).

**Table 1:** Characteristics of choking-induced cardiac arrest patients by age group

	Total	20–64 years old	65–74 years old	75–84 years old	≥85 years old	*P* value
	*N* = 268	*N* = 26	*N* = 33	*N* = 70	*N* = 139	
Age, median (IQR)	85 (79–89)	56.5 (51–62)	70 (67–72)	82 (80–83)	89 (87–92)	
Men, n (%)	144 (53.7%)	16 (61.5%)	17 (51.5%)	47 (67.1%)	64 (46.0%)	0.027
Antipsychotic use, n (%)	62 (23.1%)	15 (57.7%)	15 (45.5%)	14 (20.0%)	18 (12.9%)	< 0.001
Dopamine agonists use, n (%)	25 (9.3%)	0 (0.0%)	2 (6.1%)	13 (18.6%)	10 (7.2%)	0.017
Anticholinergic agents use, n (%)	42 (15.7%)	8 (30.8%)	7 (21.2%)	15 (21.4%)	12 (8.6%)	0.004
Sedative agents use, n (%)	80 (29.9%)	13 (50.0%)	14 (42.4%)	14 (20.0%)	39 (28.1%)	0.012
Steroid use, n (%)	13 (4.9%)	0 (0.0%)	1 (3.0%)	5 (7.1%)	7 (5.0%)	0.64
Dementia (with donepezil use), n (%)	38 (14.2%)	0 (0.0%)	3 (9.1%)	11 (15.7%)	24 (17.3%)	0.071
Stroke, n (%)	81 (30.2%)	1 (3.8%)	8 (24.2%)	28 (40.0%)	44 (31.7%)	0.002
Sjogren’s syndrome, n (%)	5 (1.9%)	0 (0.0%)	2 (6.1%)	3 (4.3%)	0 (0.0%)	0.025
Daily life independence level, n (%)						
Unrated	99 (36.9%)	25 (96.2%)	17 (51.5%)	26 (37.1%)	31 (22.3%)	< 0.001
Independence	64 (23.9%)	0 (0.0%)	9 (27.3%)	19 (27.1%)	36 (25.9%)	
Dependence	105 (39.2%)	1 (3.8%)	7 (21.2%)	25 (35.7%)	72 (51.8%)	
Nursing home, n (%)	15 (5.6%)	0 (0.0%)	1 (3.0%)	4 (5.7%)	10 (7.2%)	0.62
State of dysphagia (within 12 months)						
Dysphagia rehabilitation, n (%)	14 (5.2%)	1 (3.8%)	0 (0.0%)	4 (5.7%)	9 (6.5%)	0.58
Aspiration pneumonia, n (%)	21 (7.8%)	1 (3.8%)	1 (3.0%)	5 (7.1%)	14 (10.1%)	0.59
Procedure after cardiac arrest						
TTM, n (%)	1 (0.4%)	0 (0.0%)	0 (0.0%)	1 (1.4%)	0 (0.0%)	0.48
Tracheostomy, n (%)	13 (4.9%)	1 (3.8%)	6 (18.2%)	3 (4.3%)	3 (2.2%)	0.005
Gastrostomy, n (%)	1 (0.4%)	0 (0.0%)	0 (0.0%)	1 (1.4%)	0 (0.0%)	0.48
Dysphagia Rehabilitation, n (%)	8 (3.0%)	0 (0.0%)	1 (3.0%)	3 (4.3%)	4 (2.9%)	0.89
Survival after cardiac arrest, n (%)						
1 month following cardiac arrest, n (%)	36 (13.4%)	2 (7.7%)	9 (27.3%)	9 (12.9%)	16 (11.5%)	0.12
3 months following cardiac arrest, n (%)	15 (5.6%)	1 (3.8%)	5 (15.2%)	6 (8.6%)	3 (2.2%)	0.013
12 months following cardiac arrest, n (%)	7 (2.6%)	0 (0.0%)	3 (9.1%)	3 (4.3%)	1 (0.7%)	0.028

*Abbreviations*: *IQR* interquartile range, *TTM* targeted temperature management, *CA* cardiac arrest

**Fig. 2:**
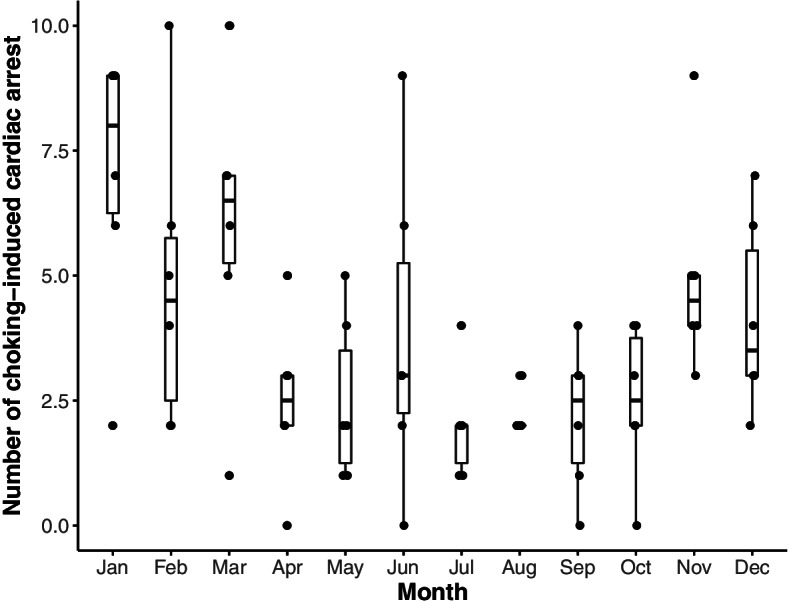
Number of choking-induced cardiac arrests by month from April 2012 to September 2018. The numbers of incidents in November to March were higher than those in the other months

We plotted the number of incident choking-induced cardiac arrest cases per month (Fig. 2). The number of incident cases in Japan was largest in winter, especially from November to March.

**Fig. 3:**
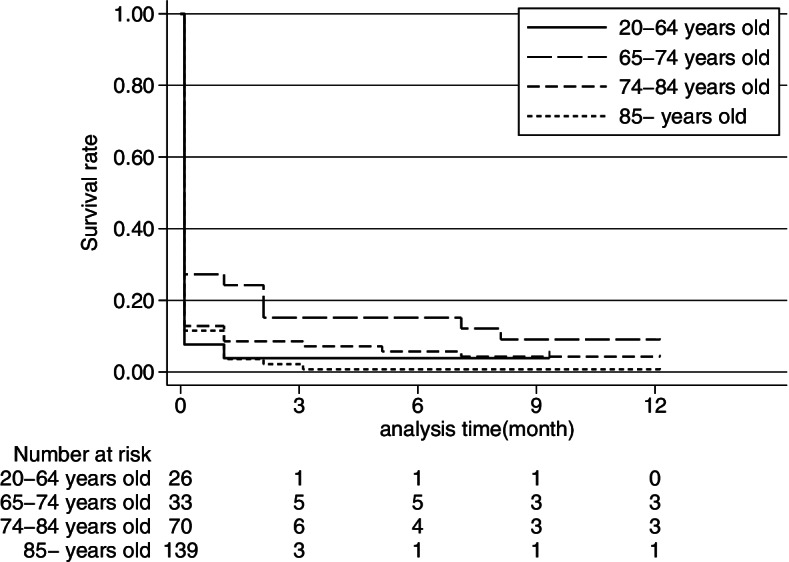
Kaplan-Meier survival curves for each age group at 12 months after choking-induced cardiac arrest. In all age groups, over 80% of choking-induced cardiac arrest patients died within 3 months after choking-induced cardiac arrest

With regard to the primary outcome, the overall 3-month survival rate was 5.9%, and the 12-month survival rate was 2.6%. Figure 3 shows the distributions of surviving patients in different age groups. In all the age groups, more than 80% of the patients died within 3 months after choking-induced cardiac arrest, and less than 10% survived to 12 months. All the patients had died by 38 months.

Of the 7 12-month survivors, 3 were on ventilators, and 5 were receiving tube feeding or TPN at 3 months. These survivors were still on ventilators and receiving tube feeding or TPN in the hospital and had not been discharged at 12 months. Only one patient (0.37%; 1/268) survived for 12 months and returned home without any assistive devices.

### Comparisons between the survivor and nonsurvivor groups at 3 months after cardiac arrest

The characteristics of survivors and nonsurvivors at 3 months after cardiac arrest are shown in Table 2. The survivors were younger in age than the nonsurvivors. All those with dysphagia rehabilitation within 12 months died. Twenty of 21 patients who had aspiration pneumonia within 12 months died within 3 months. Approximately half of the surviving patients received a tracheostomy (53.3%).

**Table 2:** Comparisons between nonsurvivors and survivors at 3 months after cardiac arrest

	Total	Nonsurvivor	Survivor	*P* value
	*N* = 268	*N* = 253	*N* = 15	
Age, median (IQR)	85 (79–89)	85 (80–89)	78 (68–84)	0.006
Men, n (%)	144 (53.7%)	136 (53.8%)	8 (53.3%)	1.00
Antipsychotic use, n (%)	62 (23.1%)	55 (21.7%)	7 (46.7%)	0.051
Dopamine agonists use, n (%)	25 (9.3%)	22 (8.7%)	3 (20.0%)	0.15
Anticholinergic agents use, n (%)	42 (15.7%)	37 (14.6%)	5 (33.3%)	0.067
Sedative agents use, n (%)	80 (29.9%)	75 (29.6%)	5 (33.3%)	0.78
Steroid use, n (%)	13 (4.9%)	12 (4.7%)	1 (6.7%)	0.54
Dementia (with donepezil use), n (%)	38 (14.2%)	36 (14.2%)	2 (13.3%)	1.00
Stroke, n (%)	81 (30.2%)	76 (30.0%)	5 (33.3%)	0.78
Sjogren’s syndrome, n (%)	5 (1.9%)	5 (2.0%)	0 (0.0%)	1.00
Daily life independence level, n (%)				
Unrated	99 (36.9%)	93 (36.8%)	6 (40.0%)	0.89
Independence	64 (23.9%)	60 (23.7%)	4 (26.7%)	
Dependence	105 (39.2%)	100 (39.5%)	5 (33.3%)	
Nursing home, n (%)	15 (5.6%)	15 (5.9%)	0 (0.0%)	1.00
State of dysphagia (within 12 months)				
Dysphagia rehabilitation, n (%)	14 (5.2%)	14 (5.5%)	0 (0.0%)	1.00
Aspiration pneumonia, n (%)	21 (7.8%)	20 (7.9%)	1 (6.7%)	1.00

*Abbreviations*: *IQR* interquartile range

### Comparisons between the ventilator-dependent and nonventilator-dependent groups at 3 months after cardiac arrest

Comparisons between the ventilator-dependent group and the nonventilator-dependent group at 3 months after cardiac arrest are shown in Table 3. Of the 15 3-month survivors, 6 were on ventilators, 9 were receiving tube feeding, and 4 were receiving central venous feeding. In the ventilator-dependent group, tracheostomy was performed in 83% (5/6), and in the nonventilator-dependent group, tracheostomy was performed in 33% (3/9). In the ventilator-dependent group, none of the patients had undergone dysphagia rehabilitation, whereas in the nonventilator-dependent group, 22% (2/9) had undergone dysphagia rehabilitation. In the ventilator-dependent group, the survival time, including the initial 3-month assessment period, was 8.0 months (IQR: 7.0–17.0 months), compared with 29.0 months (IQR: 5.0–38.0 months) in the nonventilator-dependent group. Additional file 1 shows the survival curves for each group.

**Table 3:** Comparisons between ventilator-dependent and nonventilator-dependent patients at 3 months

	Total	Nonventilator dependent	Ventilator dependent	*P* value
	*N* = 15	*N* = 9	*N* = 6	
Age, median (IQR)	78 (68–84)	78 (70–81)	76 (66–86)	0.95
Men, n (%)	8 (53%)	5 (56%)	3 (50%)	1.00
TTM, n (%)	1 (7%)	1 (11%)	0 (0%)	1.00
Tracheostomy, n (%)	8 (53%)	3 (33%)	5 (83%)	0.12
Gastrostomy, n (%)	1 (7%)	1 (11%)	0 (0%)	1.00
Dysphagia rehabilitation, n (%)	2 (13%)	2 (22%)	0 (0%)	0.49
Tube feeding at 3 months, n (%)	9 (60%)	4 (44%)	5 (83%)	0.29
Central venous catheter at 3 months, n (%)	4 (27%)	1 (11%)	3 (50%)	0.24
Survival in the 12 months, n (%)	7 (47%)	4 (44%)	3 (50%)	1.00
Survival month, median (IQR)	17 (5–29)	29 (5–38)	8 (7–17)	0.72

*Abbreviations*: *IQR* interquartile range, *TTM* targeted temperature management, *CVC* central venous catheter

## Discussion

In the present study, we revealed that the prognosis of choking-induced cardiac arrest was extremely poor when patients were not resuscitated before arrival at the hospital. Even if they achieved the ROSC and survived, they were mostly dependent on assistive devices. In addition, none of the survivors dependent on assistive devices had discontinued the use of the devices, even at the long-term follow-up. Twelve months after choking-induced cardiac arrest, only one of 268 patients returned home without any assistive devices. To our knowledge, this is the first study that showed the long-term prognosis and detailed clinical course after choking-induced cardiac arrest.

A Norwegian study of patients admitted for cardiogenic OHCA reported that the 8-year survival rate was 49% [9]. Comparatively, choking-induced cardiac arrest in this study population appears to have a markedly worse course because all the patients died within 38 months. Another previous study of OHCA, with a few cases caused by choking, showed that cognitive function and ADL in resuscitated cardiac arrest patients improved within 12 months after resuscitation [13, 14]; these results are not consistent with our results. We believe that there are two reasons for these inconsistencies. One is that the subjects in the previous study included cardiogenic cardiac arrest patients, and the other is that the subjects analyzed in our current study were choking-induced cardiac arrest patients with a relatively poor prognosis because they did not achieve the ROSC until hospital arrival. Based on the above, the prognosis of choking-induced cardiac arrest may be worse than that of cardiac arrest due to other causes. Previous studies have shown that cardiac arrest due to hypoxia is more damaging to brain neurons than cardiogenic cardiac arrest [21]. Since the process from hypoxemia to cardiac arrest also occurs in choking-induced cardiac arrest, it is pathologically reasonable that the prognosis of choking-induced cardiac arrest is worse than that of cardiogenic cardiac arrest. Therefore, cardiac arrest due to hypoxia needs to be distinguished from cardiogenic cardiac arrest.

When a patient with choking-induced cardiac arrest does not achieve the ROSC by the time he or she arrives at the hospital, the patient has likely already suffered irreversible damage, leading to an extremely poor prognosis. Only one of the 268 choking-induced cardiac arrest patients without the ROSC on hospital arrival was able to live at home 12 months after the event; this has important implications for hospital medical providers. This result suggests that the efficacy of aggressive therapeutic interventions, such as cardiopulmonary resuscitation at the hospital (intubation, intravenous fluids, chest compressions, electrical defibrillation, etc.), for choking-induced cardiac arrest may be low. These findings support that failure to achieve the ROSC before arrival at the hospital is in itself a strong prognostic factor for a poor outcome [22, 23].

It is generally understood that early removal of choking objects in choking-induced cardiac arrest patients contributes significantly to the ROSC and an improvement in prognosis [7, 24]. Considering this result, it is suggested that to improve the prognosis of choking-induced cardiac arrest, choking objects need to be removed and resuscitation needs to be performed before arrival at the hospital. In the future, it is necessary to research prehospital asphyxia interventions (Heimlich maneuver, intubation, etc.) and effective ways to educate community members about providing prehospital asphyxia interventions.

Furthermore, this study clarified that age, psychiatric disorders, Parkinson’s symptoms, and Sjogren’s syndrome were observed more frequently in these choking-induced cardiac arrests. If families and caregivers are aware of these factors beforehand, they can prevent choking and provide prompt assistance when choking occurs. Aging has been reported to increase the risk of dysphagia [25], and the number of choking-induced cardiac arrests in this study was highest in the ≥85 age group. We believe that the increased prevalence of aspiration pneumonia and history of dysphagia rehabilitation with age is an indication that swallowing function declines with age. In contrast, most of the choking-induced cardiac arrests in the young age group had no history of aspiration pneumonia or dysphagia rehabilitation. Additional characteristics of the young age group were high rates of antipsychotic and sedative agents use. This indicates that the presence of psychiatric disorders may have a stronger relationship with choking-induced cardiac arrest than dysphagia in the young age group. This may be due to the possibility that medication use and psychiatric disorders pose risks for choking-induced cardiac arrest. With regard to medications, extrapyramidal side effects of antipsychotic agents may increase the risk of choking [26, 27]. With regard to psychiatric disorders, it has been reported that patients with a history of psychiatric disorders have a tendency to eat quickly and consume excessive amounts of food, which may lead to choking [28]. In addition, Parkinson’s disease and Sjogren’s syndrome have been reported to cause dysphagia [28, 29] and may be risk factors for choking-induced cardiac arrest. Particularly, in this study, 1.9% of patients with choking-induced cardiac arrest had been diagnosed with Sjogren’s syndrome. However, the incidence of Sjogren’s syndrome has been reported to be 0.05 to 0.1% in previous studies [30, 31], and the incidence in this study is higher than those in previous studies. To the best of our knowledge, this is a new finding in this study. Regarding seasonality, the incidence of choking-induced cardiac arrest in Japan tended to be higher in winter (November to March). This is a characteristic of Japan and has not been observed in Western countries [1]. Choking is influenced by dietary culture and habits, and choking objects regionally [2, 32]. According to previous studies, the high incidence of choking in winter in Japan may be due to the consumption of rice cakes [6, 33]. If the increase in choking-induced cardiac arrest from November to March is considered preventable, 1/3 of the annual number of cases can be prevented.

The strength of this study was the long follow-up period. Specifically, we were able to follow patients for up to 38 months. Furthermore, since we used claims data, we were able to monitor the dependence on assistive devices over a long period of time. Previous studies on choking-induced cardiac arrest have reported only one-month survival rates and survival discharge rates, but in this study, we found that even those who survived for a long time after choking-induced cardiac arrest were mostly dependent on assistive devices. Essentially, in deciding on a treatment plan, the survival rate as well as the living conditions after discharge from the hospital should be taken into consideration. In this study, we were able to show the survival rate and the degree of dependence on devices in long-term survivors. This information is meaningful for the patients’ families and doctors.

### Limitations

The present study has some limitations. First, we could not assess some detailed clinical information; for example, witnessed arrest, bystander cardiopulmonary resuscitation (CPR), initial rhythm, and the ROSC on site. In addition, information about the patient’s address and the medical institution to which the patient was transported was not available. The Shizuoka Kokuho Database does not include information about the time, place, and caregiver at the onset of choking-induced cardiac arrest. Second, choking-induced cardiac arrest without resuscitation at the hospital was not included in this analysis. The prognosis of those who achieved the ROSC before hospital transport could be better, and it might be difficult to compare this result with other results of choking-induced cardiac arrest. Third, this database consisted of LEHI and NHI and they are different from insurance in the general population. Individuals with these types of health insurance plans may be different from the general population. Therefore, it is difficult to generalize the results of this study.

## Conclusions

The prognosis of choking-induced cardiac arrest was extremely poor when patients were not resuscitated before arrival at the hospital. Those who survived were mostly dependent on assistive devices. In addition, none of the survivors dependent on assistive devices discontinued the use of the devices, even after long-term follow-up.

## Supplementary Information

**Additional file 1.**

## Data Availability

The data that support the findings of this study are available from the Research Support Center in Shizuoka General Hospital; however, restrictions apply to the availability of these data, which were used under license for the current study and are not publicly available. Data are, however, available from the corresponding authors upon reasonable request and with permission of the Research Support Center in Shizuoka General Hospital.

## Abbreviations

CPC: Cerebral performance category
OHCA: Out-of-hospital cardiac arrest
ADL: Activities of daily living
NHI: National Health Insurance
LEHI: Late Elder’s Health Insurance
EHI: Employee’s Health Insurance
ICD-10: 10th Revision of the International Classification of Diseases
ATC: Anatomical Therapeutic Chemical Classification System
ROSC: Return of spontaneous circulation
TPN: Total parenteral nutrition
EMS: Emergency medical services
SD: Standard deviation
IQR: Interquartile range
TTM: Targeted temperature management
CA: Cardiac arrest
CVC: Central venous catheter
CPR: Cardiopulmonary resuscitation

## References

[1] Pavitt MJ, Nevett J, Swanton LL, Hind MD, Polkey MI, Green M (2017). London ambulance source data on choking incidence for the calendar year 2016: an observational study. BMJ Open Respir Res.

[2] Landoni G, Morselli F, Silvetti S, Frontera A, Zangrillo A, De Domenico P (2020). Pizza in adults and grape in children are the most frequent causes of foreign body airway obstruction in Italy. A national media-based survey. Resuscitation.

[3] Vital Statistics Vital statistics of Japan Final data General mortality Volume 1 5–30 Trends in deaths and death rates (per 100,000 population) from accidents by external causes (the list of three-character categories):Japan Yearly 2019 | File | Browse Statistics | Portal Site of Official Statistics of Japan n.d. https://www.e-stat.go.jp/en/stat-search/files?page=1&layout=datalist&toukei=00450011&tstat=000001028897&cycle=7&year=20190&month=0&tclass1=000001053058&tclass2=000001053061&tclass3=000001053065&stat_infid=000031982769&result. Accessed 9 Aug 2021.

[4] Deaths in the Home and Community by Age Group and Cause - Injury Facts n.d. https://injuryfacts.nsc.org/home-and-community/home-and-community-overview/deaths-in-the-home-and-community-by-age-group-and-cause/. Accessed 9 Aug 2021.

[5] Chung C-H, Lai C-H, Chien W-C, Lin C-H, Cheng C-H (2013). A population-based study of inpatients admitted due to suffocation in Taiwan during 2005-2007. Accid Anal Prev.

[6] Kiyohara K, Sakai T, Nishiyama C, Nishiuchi T, Hayashi Y, Iwami T (2018). Epidemiology of Out-of-Hospital Cardiac Arrest Due to Suffocation Focusing on Suffocation Due to Japanese Rice Cake: A Population-Based Observational Study From the Utstein Osaka Project. J Epidemiol.

[7] Igarashi Y, Yokobori S, Yoshino Y, Masuno T, Miyauchi M, Yokota H (2017). Prehospital removal improves neurological outcomes in elderly patient with foreign body airway obstruction. Am J Emerg Med.

[8] Suematsu Y, Zhang B, Kuwano T, Sako H, Ogawa M, Yonemoto N (2019). Citizen bystander-patient relationship and 1-month outcomes after out-of-hospital cardiac arrest of cardiac origin from the All-Japan Utstein Registry: a prospective, nationwide, population-based, observational study. BMJ Open.

[9] Kvakkestad KM, Sandvik L, Andersen GØ, Sunde K, Halvorsen S (2018). Long-term survival in patients with acute myocardial infarction and out-of-hospital cardiac arrest: A prospective cohort study. Resuscitation.

[10] Andrew E, Nehme Z, Wolfe R, Bernard S, Smith K (2017). Long-term survival following out-of-hospital cardiac arrest. Heart.

[11] Wachelder EM, Moulaert VRMP, van Heugten C, Verbunt JA, Bekkers SCAM, Wade DT (2009). Life after survival: long-term daily functioning and quality of life after an out-of-hospital cardiac arrest. Resuscitation.

[12] Green CR, Botha JA, Tiruvoipati R (2015). Cognitive function, quality of life and mental health in survivors of our-of-hospital cardiac arrest: a review. Anaesth Intensive Care.

[13] Ørbo M, Aslaksen PM, Larsby K, Schäfer C, Tande PM, Anke A (2016). Alterations in cognitive outcome between 3 and 12 months in survivors of out-of-hospital cardiac arrest. Resuscitation.

[14] Moulaert VRM, van Heugten CM, Gorgels TPM, Wade DT, Verbunt JA (2017). Long-term outcome after survival of a cardiac arrest: a prospective longitudinal cohort study. Neurorehabil Neural Repair.

[15] Nishimura S, Kumamaru H, Shoji S, Nakatani E, Yamamoto H, Ichihara N, et al. Assessment of coding-based frailty algorithms for long-term outcome prediction among older people in community settings: a cohort study from the Shizuoka Kokuho Database. Age Ageing. 2022;51. 10.1093/ageing/afac009. 10.1093/ageing/afac009 PMC9077119 35231096

[16] Shimada K, Yamamoto H, Nakatani E, Kumamaru H, Nishimura S, Ichihara N (2021). Real-World Evidence of the Incidence of and Risk Factors for Type 1 Diabetes Mellitus and Hypothyroidism as Immune-Related Adverse Events Associated With Programmed Cell Death-1 Inhibitors. Endocr Pract.

[17] Kohsaka S, Kumamaru H, Nishimura S, Shoji S, Nakatani E, Ichihara N (2021). Incidence of adverse cardiovascular events in type 2 diabetes mellitus patients after initiation of glucose-lowering agents: A population-based community study from the Shizuoka Kokuho database. J Diabetes Investig.

[18] Nakatani E, Tabara Y, Sato Y, Tsuchiya A, Miyachi Y. Data resource profile of Shizuoka Kokuho Database (SKDB) using integrated health- and care-insurance claims and health checkups: the Shizuoka Study. J Epidemiol. 2021. 10.2188/jea.JE20200480. 10.2188/jea.JE20200480 PMC9263618 33518592

[19] de Graaf C, Donders DNV, Beesems SG, Henriques JPS, Koster RW (2021). Time to return of spontaneous circulation and survival: when to transport in out-of-hospital cardiac arrest?. Prehosp Emerg Care.

[20] Tang KT, Hsieh MH (2010). A case of schizophrenia with dysphagia successfully treated by a multidimensional approach. Gen Hosp Psychiatry.

[21] http://www.ilcjapan.org/linksE/doc/Overview_of_the_Revision_of_LTCI.pdf. Accessed 10 Oct 2021.

[22] https://www.mhlw.go.jp/english/policy/care-welfare/care-welfare-elderly/dl/ltcisj_e.pdf. Accessed 10 Oct 2021.

[23] Uray T, Lamade A, Elmer J, Drabek T, Stezoski JP, Missé A (2018). Phenotyping cardiac arrest: bench and bedside characterization of brain and heart injury based on etiology. Crit Care Med.

[24] Nagao K, Nonogi H, Yonemoto N, Gaieski DF, Ito N, Takayama M (2016). Duration of prehospital resuscitation efforts after out-of-hospital cardiac arrest. Circulation.

[25] Igarashi Y, Norii T, Sung-Ho K, Nagata S, Tagami T, Femling J (2019). New classifications for Life-threatening foreign body airway obstruction. Am J Emerg Med.

[26] Soroudi A, Shipp HE, Stepanski BM, Ray LU, Murrin PA, Chan TC (2007). Adult foreign body airway obstruction in the prehospital setting. Prehosp Emerg Care.

[27] Nishikubo Kaori, Mise Kazuyo, Ameya Misato, Hirose Kahori, Kobayashi Taisuke, Hyodo Masamitsu (2015). Quantitative evaluation of age-related alteration of swallowing function: Videofluoroscopic and manometric studies. Auris Nasus Larynx.

[28] Stewart JT (2018). Covert dysphagia and recurrent pneumonia related to antipsychotic treatment. J Psychiatry Neurosci.

[29] Taniguchi Y, Iwagami M, Sakata N, Watanabe T, Abe K, Tamiya N (2021). Epidemiology of food choking deaths in japan: time trends and regional variations. J Epidemiol.

[30] Kulkarni DP, Kamath VD, Stewart JT (2017). Swallowing disorders in schizophrenia. Dysphagia.

[31] Pierce Jenny L., Tanner Kristine, Merrill Ray M., Miller Karla L., Kendall Katherine A., Roy Nelson (2016). Swallowing Disorders in Sjögren’s Syndrome: Prevalence, Risk Factors, and Effects on Quality of Life. Dysphagia.

[32] Tsuboi H, Asashima H, Takai C, Hagiwara S, Hagiya C, Yokosawa M (2014). Primary and secondary surveys on epidemiology of Sjögren’s syndrome in Japan. Mod Rheumatol.

[33] Mavragani CP, Moutsopoulos HM (2010). The geoepidemiology of Sjögren’s syndrome. Autoimmun Rev.

