# Correction: Long-term prognosis and clinical course of choking-induced cardiac arrest in patients without the return of spontaneous circulation at hospital arrival: a population-based community study from the Shizuoka Kokuho Database

**DOI:** 10.1186/s12873-022-00701-w

**Published:** 2022-09-07

**Authors:** Takahiro Miyoshi, Hideki Endo, Hiroyuki Yamamoto, Koki Shimada, Hiraku Kumamaru, Nao Ichihara, Yoshiki Miyachi, Hiroaki Miyata

**Affiliations:** 1 Shizuoka Graduate University of Public Health, 4-27-2 Kita Ando Aoi-ku, Shizuoka City, 420-0881 Japan; 2 Department of Health Policy and Management, School of Medicine, Keio University, Tokyo, Japan; 3 Department of Healthcare Quality Assessment, The University of Tokyo Graduate School of Medicine, Tokyo, Japan

**Correction: BMC Emerg Med 22, 120 (2022)**

**https://doi.org/10.1186/s12873-022-00676-8**

The original article [1] contained errors in the order of the references in the article which have since been corrected.
