# Supplementary material for: Long-term prognosis and clinical course of choking-induced cardiac arrest in patients without the return of spontaneous circulation at hospital arrival: a population-based community study from the Shizuoka Kokuho Database
Source: BMC Emerg Med. 2022 Jul 6;22:120. doi: 10.1186/s12873-022-00676-8 (PMC9258190; doi:10.1186/s12873-022-00676-8)

Additional file 1(Additional file 1.pdf)

Kaplan-Meier survival curves of nonventilator-dependent and ventilator-dependent survivors at 3 months. The survival time was longer in those independent of ventilator support.

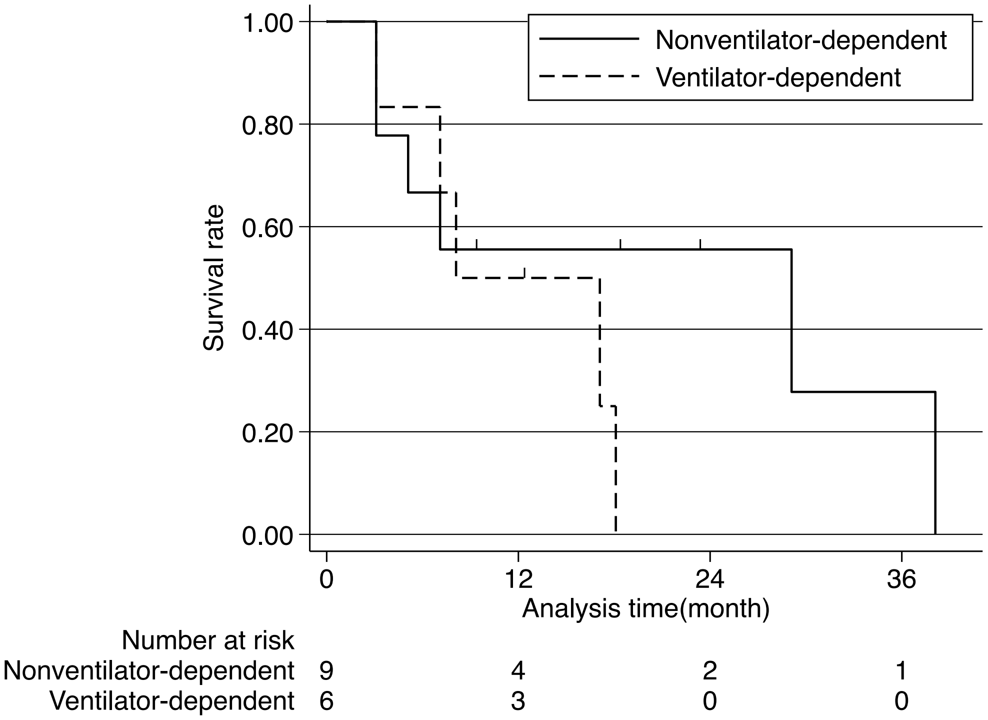

Supplement: Supplementary file 1 — Additional file 1. [file 12873_2022_676_MOESM1_ESM.pdf]
